# Human Rad51 mediated DNA unwinding is facilitated by conditions that favour Rad51-dsDNA aggregation

**DOI:** 10.1186/1471-2091-10-2

**Published:** 2009-01-09

**Authors:** Kamakshi Balakrishnan, Neeraja M Krishnan, Anagha Kulkarni, Basuthkar J Rao

**Affiliations:** 1 Department of Biological Sciences, Tata Institute of Fundamental Research, Homi Bhabha Road, Colaba, Mumbai-400 005, India

## Abstract

**Background:**

Human Rad51 (RAD51), analogous to its bacterial homolog, RecA, binds and unwinds double stranded DNA (dsDNA) in the presence of certain nucleotide cofactors. ATP hydrolysis is not required for this process, because even ATP non hydrolysable analogs like AMP-PNP and ATPγS, support DNA unwinding. Even ADP, the product of ATP hydrolysis, feebly supports DNA unwinding.

**Results:**

We find that human Rad52 (RAD52) stimulates RAD51 mediated DNA unwinding in the presence of all Adenine nucleotide cofactors, (except in AMP and no nucleotide conditions that intrinsically fail to support unwinding reaction) while enhancing aggregation of RAD51-dsDNA complexes in parallel. Interestingly, salt at low concentration can substitute the role of RAD52, in facilitating aggregation of RAD51-dsDNA complexes, that concomitantly also leads to better unwinding.

**Conclusion:**

RAD52 itself being a highly aggregated protein perhaps acts as scaffold to bring together RAD51 and DNA molecules into large co-aggregates of RAD52-RAD51-DNA complexes to promote RAD51 mediated DNA unwinding reaction, when appropriate nucleotide cofactors are available, presumably through macromolecular crowding effects. Our work highlights the functional link between aggregation of protein-DNA complexes and DNA unwinding in RAD51 system.

## Background

RAD51 recombinase, the eukaryotic homolog of RecA, is the core component in homologous recombination (HR). It performs fundamental roles such as homologous pairing and strand exchange in repairing DNA double strand breaks [1,2]. Rad51 binds both ssDNA/dsDNA [3-5] and forms right-handed helical nucleoprotein filaments in which DNA is extended 1.5 times that of B form DNA [4,6-8]. Such extended conformation of DNA in ssDNA-protein filaments is instrumental in facilitating homology search and strand exchange in a three stranded pairing system [8-10]. Even though the equivalent scenario of extended DNA configuration is demonstrable in dsDNA-protein filament, its relevance in HR is not fully understood. However, based on *E. coli *RecA system, it is surmised that unwinding associated with dsDNA-protein filament is similarly important in four-stranded pairing and strand exchange processes [11]. The first and also most recent report that describes four-stranded exchange reaction catalyzed by RAD51 strongly underscores this aspect where RAD51-dsDNA filament is shown to engage in duplex-duplex pairing [12]. The current study is aimed to better understand the functions of dsDNA-protein filament. Directly impinging on this issue is the relevance of nucleotide cofactor, ATP. The current understanding of binding/hydrolysis of ATP by RAD51 with respect to its HR function is still evolving, and the same is different from that of RecA [13,14]. RAD51 possesses DNA dependent ATPase activity, which is largely stimulated by ssDNA and to some lesser extent by dsDNA [4,7,10]. ATP hydrolysis is critical for homology directed DNA repair *in vivo *[15] as well as in some *in vitro *conditions [10,16], but not in some other conditions [17,18], implying that RAD51 system is endowed with sufficient dynamicity that it can bring about various functional outcomes based on given reaction conditions. Inspired by this property of the protein system, our current study addresses dsDNA unwinding reaction by RAD51 with respect to not only nucleotide cofactor conditions, but also the role of RAD52, an important regulator of RAD51 functions, in the same.

Rad52 interacts genetically/physically with Rad51 and stimulates a variety of its functions during HR [19-22]. Both proteins co-localize in distinct repair proficient nuclear foci in response to DNA damage [23]. Rad52 is required for such foci formation during meiosis [24]. Mechanistically, it has been shown that in *Saccharomyces cerevisiae*, Rad52 is critical for recruiting Rad51 to repair sites, *in vivo *[25]. Chromatin immunoprecipitation assays demonstrate Rad52 requirement for recruitment of yeast Rad51 to HO induced repair sites at MATa locus [26,27]. Biochemically, Rad52 stimulates strand exchange activity of Rad51 [28-30], is shown to displace RPA [31,32], stabilize the Rad51-ssDNA filament [33]. In light of such demonstrated functions of Rad52 in a variety of Rad51 activities, we realized the importance of probing such collaborative relationship in RAD51 mediated dsDNA unwinding reaction.

It is relevant to point out here that RAD51 is one of the versatile DNA binding proteins in the cell whose ability to laterally diffuse on dsDNA as studied by Total Internal Reflection Microscopy is not only rather fast but also does not seem to need the energy of ongoing ATP hydrolysis [34]. Here we show that such a dynamic protein system brings about efficient unwinding of dsDNA in conditions that do not hydrolyze ATP, a result consistent with similar findings reported earlier by Chi et al, 2006 [17]. Importantly, our current study shows that conditions which facilitate better aggregation of RAD51-dsDNA also stimulate steady-state level of dsDNA unwinding in parallel. These are achieved by either presence of RAD52 or salt (KCl) at low concentration, where macromolecular crowding effects facilitate RAD51 mediated DNA unwinding reaction.

## Results

RAD51 unwinds dsDNA [4,17], but the underlying mechanism of this process with respect to biochemical reaction conditions is far from clear. We have addressed this aspect in our current study. Here we delineate specific nucleotide cofactor requirements for DNA unwinding and provide mechanistic insights on how RAD52 collaborates with RAD51 in this process. Furthermore, we show that unwinding of duplex DNA by RAD51 correlates positively with aggregation of RAD51-dsDNA complexes.

### RAD51 mediated dsDNA unwinding does not require ATP hydrolysis

**Figure 1:**
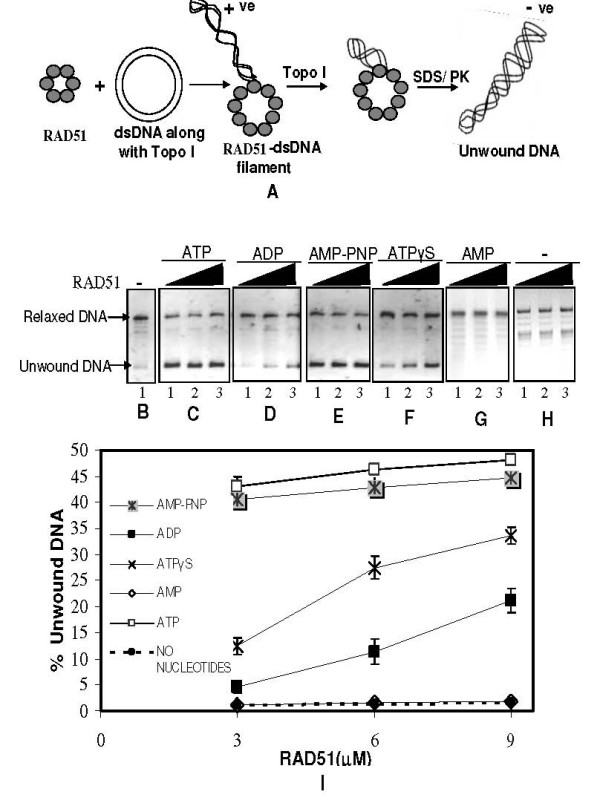
**A) Schematic for DNA unwinding assay**. Image modified from Chi et al (Fig. 4A) [17]. RAD51 is incubated with relaxed dsDNA along with Topo I. RAD51 upon binding to dsDNA causes dsDNA unwinding generating compensatory overwinding elsewhere in the same DNA molecule. Topo I acts as a "swivel" releasing excess turns thereby capturing the events of DNA unwinding that can be monitored after deproteinization by agarose gel electrophoresis. **(B-I) RAD51 unwinds dsDNA in presence of various nucleotide cofactors**. Relaxed φX174DNA (30 μM) along with Topo I was incubated with increasing concentrations of RAD51 (3 μM, 6 μM & 9 μM) (lanes 1–3 in Panels C – H) in binding buffer R [30 mM Tris-HCl (pH 7.6), 1 mM DTT, 1 mM MgCl_2_] at 37°C for 12 minutes, in presence of either ATP, ADP, AMP-PNP, ATPγS, or AMP (1 mM each) (Panels C – G) or no nucleotide (Panel H), followed by deproteinization and agarose gel assay (Methods). Panel B represents no protein control. Intensity of DNA bands was quantified using Image J software. Percentage unwound DNA (background-corrected and quantified as explained in Materials and methods) was expressed as a ratio of the intensity associated with unwound DNA band to the sum of intensities corresponding to unwound as well as relaxed dsDNA bands and plotted as a function of RAD51 concentration (Panel I). Error bars denote standard deviation in data values across three independent experiments. No significant variation was observed between experiments (2 tailed t-test, P < 0.05).

Fully relaxed circular dsDNA was generated from φX174 supercoiled plasmid by the action of *Drosophila *Topo I. Subsequent unwinding of relaxed dsDNA by RAD51 was assayed in the presence of Topo I, followed by deproteinization and agarose gel electrophoresis (Methods). DNA unwinding as a function of RAD51 concentration depended entirely on nucleotide cofactor conditions. ATP as well as its poorly/non-hydrolysable analogues such as ATPγS/AMP-PNP facilitated efficient unwinding (lanes 1–3, Panels C, F & E, Fig. 1), while the absence of nucleotide cofactor failed in the same (lanes 1–3, Panel H, Fig. 1). Surprisingly, detectable level of unwinding was observed even in presence of ADP (lanes 1–3, Panel D, Fig. 1), whereas with AMP no unwinding was observed (lanes 1–3, Panel G, Fig. 1). Further quantification of the gel revealed, maximal DNA unwinding (~44%) even at the lowest RAD51 concentration [3 μM] in the presence of ATP/AMP-PNP, whereas DNA unwinding increased as a function of RAD51 concentration in ATPγS/ADP conditions and reached about 34% and 22% respectively at maximal protein concentration (9 μM) (Fig. 1I). Thus unwinding appears to be ATP binding but not hydrolysis dependent, as not only ATP but also its non-hydrolysable analogs support the process, which is in congruence with previously published reports [4] and [17]. However, even product of ATP hydrolysis, ADP was seen to support DNA unwinding by RAD51, a result that appears somewhat counter-intuitive at the outset. But in fact, even this observation is consistent with earlier result that RAD51 binds ssDNA sufficiently well that it promotes strand annealing even in ADP conditions, a property starkly contrasting with that of well studied *E. coli *RecA protein [35]. Furthermore, preliminary linear dichroism experiments with RAD51, calf-thymus dsDNA and ADP have confirmed that the HsRad51-dsDNA complex is also stable in the presence of ADP (H.K. Kim, K. Morimatsu & B. Nordén, unpublished observations). Thus, ADP stabilizes both ssDNA and dsDNA complex formation with HsRad51, although it does not promote the strand exchange reaction. This could suggest that the role of the nucleotide cofactors is not only to stabilize or destabilize the protein-DNA complex, but also involves changing the conformation of the complex to promote the strand exchange reaction. In order to eliminate the possibility that unwinding observed in ADP condition is not a trivial consequence of either ATP contamination or artifactual conversion of ADP to ATP, we assessed the integrity of ADP by Thin Layer Chromatography (TLC) assay [36]. Expectedly we found that ADP containing samples showed no traces of ATP contamination. Added ADP was pure and no artifactual conversion to ATP had happened (data not shown). Therefore we strongly believe that ADP effects were genuine.

### RAD52 stimulates RAD51 mediated duplex unwinding

**Figure 2:**
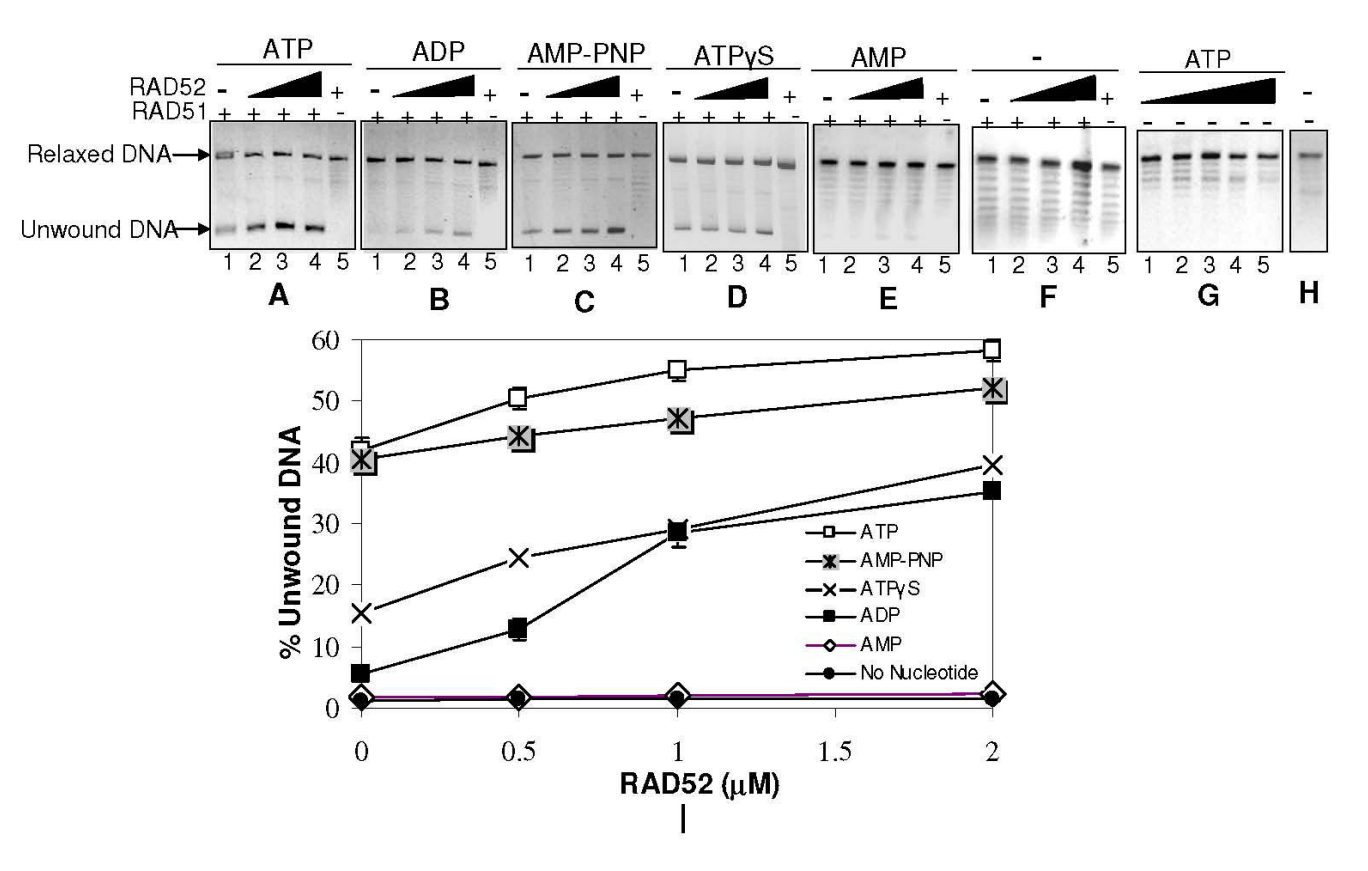
**RAD52 stimulates RAD51 mediated dsDNA unwinding**. Relaxed φX174 DNA (30 μM) along with Topo I was incubated with RAD51 (3.0 μM) in presence of increasing concentration of RAD52 (0 μM, 0.5 μM, 1.0 μM & 2.0 μM) (lanes 1–4 in each Panel) in binding buffer R at 37°C for 12 minutes, in presence of either ATP, ADP, AMP-PNP, ATPγS, or AMP (1 mM each) (Panels A-E) or no nucleotide (Panel F), followed by deproteinization and agarose gel assay (Methods). Lane 5 in all the Panels has all the components except RAD51. Panel G represents only RAD52 control in presence of ATP. Panel H represents no protein control. Percentage unwound DNA (normalized and quantified) was expressed as a ratio of the intensity associated with unwound DNA band to the sum of intensities corresponding to unwound as well as relaxed dsDNA bands. This was plotted as a function of RAD52 concentration (Panel I). Data points were statistically analyzed as explained earlier.

**Figure 3:**
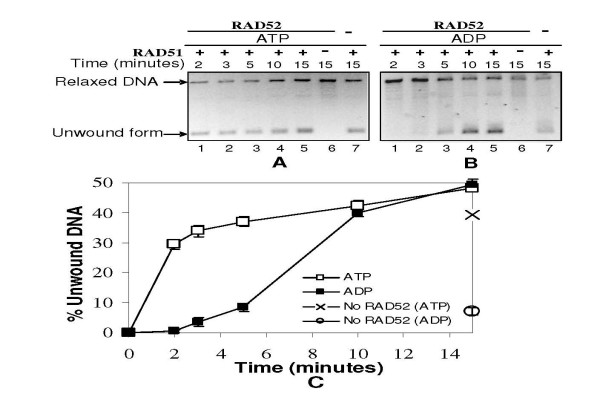
**RAD52 stimulates RAD51 mediated dsDNA unwinding even in presence of ADP: Time-course analysis**. Relaxed φX174 DNA (30 μM) along with Topo I was incubated with RAD51 (3.0 μM) in presence of RAD52 (2.0 μM) in binding buffer R at 37°C with either 1 mM ATP (Panel A) or ADP (Panel B). Reactions were terminated after indicated time points by SDS/Proteinase K treatment and analyzed on agarose gels. Lane 6 in both Panels represents only RAD52 control while lane 7 represents only RAD51 control. Percentage intensity of unwound DNA to total DNA per lane (as explained earlier) was plotted as a function of time (Panel C).

In order to test the role of RAD52 in DNA unwinding, we titrated the reactions with increasing levels of RAD52 while maintaining RAD51 concentration constant at a lower level. As expected, conditions that failed to promote RAD51 mediated unwinding, namely absence of nucleotide cofactor or presence of AMP, remained ineffective even with addition of RAD52 (lanes 2–4, Panels F & E, Fig. 2). On the other hand, nucleotide cofactor conditions that are congenial for RAD51 mediated unwinding, namely ATP/AMP-PNP/ATPγS/ADP, revealed marginal and detectable improvement in unwinding following RAD52 addition (lanes 2–4, Panels A, C, D & B, Fig. 2), while RAD52 alone did not lead to any detectable unwinding in the presence or absence of any of these nucleotide cofactors (lane 5, Panels A-F & lanes 1–3, Panel G, Fig. 2). Moreover, such RAD52 mediated enhancement of DNA unwinding by RAD51 was evident even in presence of ADP, when time-course of unwinding was studied. At fixed concentrations of RAD51 and RAD52, unwinding efficiency with ADP, reached a level similar to that of ATP, within 10 minutes of incubation (lane 4, Panel B, Fig. 3) (Fig. 3C). On the contrary, ADP containing reaction that lacked RAD52, showed only marginal unwinding which hardly increased with time (only last time-point is shown; lane 7, Panel B, Fig. 3) (Fig. 3C).

### RAD52 forms large RAD52-RAD51-DNA complexes

**Figure 4:**
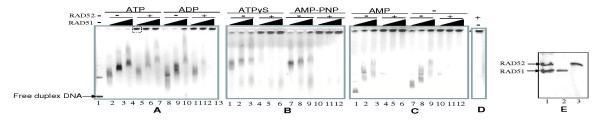
**RAD52 promotes formation of large complexes of RAD52-RAD51-DNA: gel shift assay**. Supercoiled φX174 DNA (30 μM) was incubated with increasing concentrations of RAD51 (3 μM, 6 μM & 9 μM) either in presence or absence of RAD52 (2 μM) in binding buffer R at 37°C for 12 minutes, in presence of either ATP, ADP, ATPγS, AMP-PNP, or AMP (1 mM each) or no nucleotide, followed by agarose gel assay (Methods). Controls (Lane 1 in Panel A: no proteins; Panel D: no RAD51). Lane 5 in Panel A was excised (from the region shown in thatched box) and subjected to analyses on 12% SDS-PAGE gel followed by Coomassie staining (lane 1, Panel E). Purified RAD51 (lane 2, Panel E) and RAD52 proteins (lanes 3, Panel E).

To understand molecular basis of RAD52 mediated stimulation on DNA unwinding by RAD51, we carried out DNA binding studies of RAD51 and RAD52 with circular dsDNA in the presence of various nucleotide cofactors. RAD51 protein when bound to dsDNA, generated distinctly slower migrating gel-shifted complexes as a function of increasing protein concentration. This was so in all nucleotide conditions, suggesting RAD51 binding to dsDNA was not dependent on nucleotide cofactor conditions (lanes 2–4 & 8–10, Panel A: lanes 1–3 & 7–9, Panels B & C, Fig. 4) which is consistent with previously published reports [17]. Under same conditions, binding of RAD52 alone to dsDNA generated large protein-DNA complexes that failed to enter the gel (Panel D, Fig. 4) as reported earlier [37]. Interestingly, we find that, addition of RAD52 converted RAD51-DNA gel-shifted complexes also into large complexes that fail to enter the gel (lanes 5–7 & 11–13, Panel A: lanes 4–6 and 10–12, Panels B & C, Fig. 4). Irrespective of the nucleotide cofactor conditions, addition of RAD52 resulted in large RAD52-RAD51-DNA complexes. We have further confirmed that these large protein-DNA complexes entrapped in the wells indeed contain both RAD51 and RAD52 proteins by excising agarose gel fragments containing these complexes, and subjecting them to protein compositional analyses by SDS-PAGE (Data shown only for complex formed in ATP conditions, the boxed region of lane 5, analyzed in lane 1, Panel E, Fig. 4).

### RAD52 co-aggregates with RAD51 on DNA

**Figure 5:**
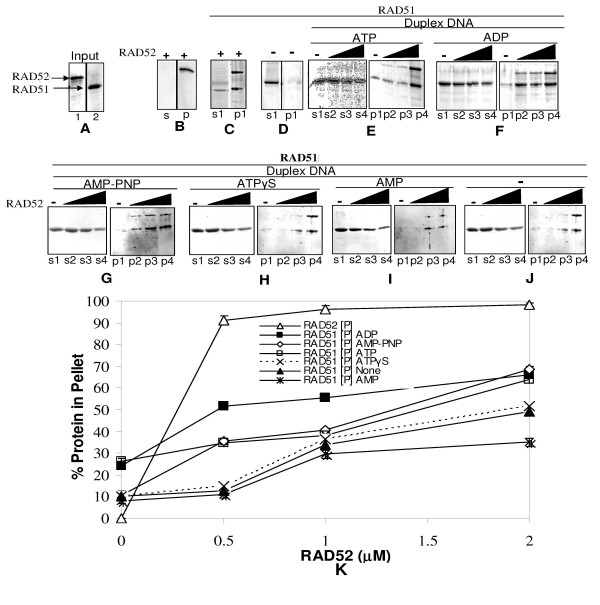
**RAD52 promotes coaggregation of RAD52-RAD51-DNA complexes: centrifugation assay followed by protein analyses**. Relaxed φX174 DNA (30 μM) along with Topo I was incubated with RAD51 (3.0 μM) in presence of increasing concentration of RAD52 (0 μM, 0.5 μM, 1.0 μM & 2.0 μM) in binding buffer R at 37°C for 12 minutes, in presence of either ATP, ADP, AMP-PNP, ATPγS, or AMP (1 mM each) (Panels E-I) or no nucleotide (Panel J), followed by centrifugation assay (Methods) and analyses of proteins by SDS-PAGE (Methods). Lanes s1–s4 and p1–p4 in all the Panels represent supernatant and pellet fractions for increasing concentrations of RAD52 respectively. Lanes 1 & 2 of Panel A show input RAD52 and RAD51 respectively used in centrifugation assay. No DNA controls: lanes s1 and p1 of Panel C correspond to supernatant and pellet fractions for RAD51 (3 μM) and RAD52 (2 μM) together, while the same for Panels B and D correspond to RAD52 protein alone and RAD51 protein alone, respectively. Intensity of protein bands was quantified using Image J software. Percentage of RAD51 protein in pellet was expressed as a ratio of the total intensity associated with pellet and supernatant protein bands and plotted as a function of RAD52 protein concentration (Panel K) (percentage of protein in supernatant fraction will therefore be 100% minus pellet fraction). The quantitative data has been given for RAD52 corresponding to only ATP set.

**Figure 6:**
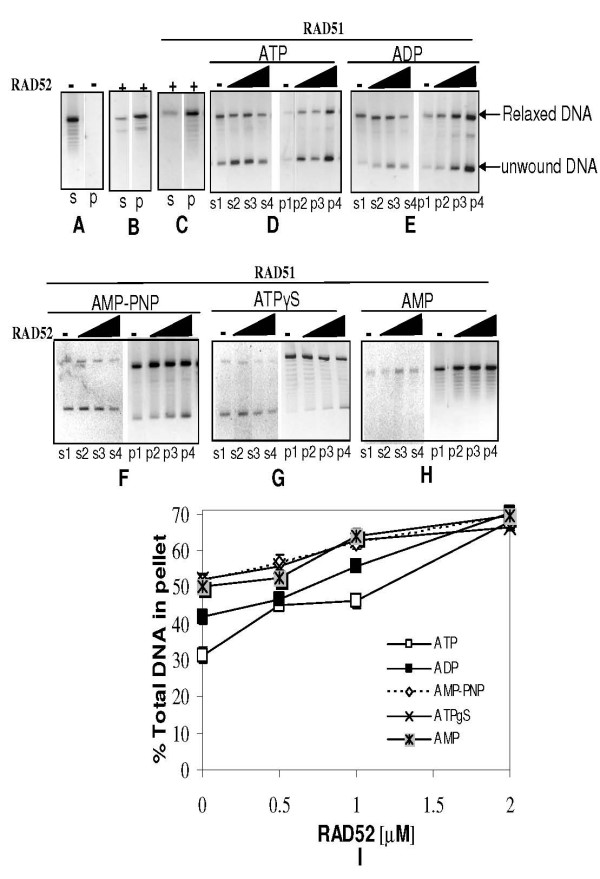
**RAD52 promotes coaggregation of RAD52-RAD51-DNA complexes: centrifugation assay followed by DNA analysis**. Centrifugation assay was carried out as explained in legend for Fig. 5. Panels (D-H) correspond to same samples described in Fig. 5, except that samples were deproteinized for DNA analyses (Methods). Controls: Lanes s and p of Panels A, B & C correspond to supernatant and pellet fractions for no proteins, RAD52 (2 μM) and [RAD51 (3 μM) plus RAD52 (2 μM)] controls respectively (all containing DNA without any nucleotide cofactors). Percentage of DNA (relaxed and unwound forms) in pellet fraction was expressed as a ratio of the total DNA (relaxed and unwound forms) in both pellet and supernatant fractions and plotted as a function of RAD52 concentration (Panel I).

In order to corroborate that these complexes indeed house both proteins and DNA, we carried out centrifugation assay and tested the ability of RAD52 to "co-aggregate" both RAD51 and itself onto DNA. Also, the same was analyzed with respect to ongoing DNA unwinding reaction. RAD51 protein, under the current experimental conditions, predominantly stayed in supernatant while a minor fraction sedimented following centrifugation assay (lane s1, Panel D, Fig. 5). On the contrary, RAD52 protein that is largely in aggregated state by itself (lane p, Panel B, Fig. 5), increases aggregation prone ness of RAD51 protein in its presence (lanes p1 and s1, Panel C, Fig. 5) where RAD52 protein retains its high state of aggregation (lane p1, Panel C, Fig. 5). Furthermore, we find RAD51-dsDNA complexes that are largely soluble in ATP/ADP conditions (lanes s1, Panels E & F, Fig. 5) get aggregated with addition of RAD52. This is evidenced by increase in levels of RAD51 protein (lanes p2–p4, Panels E & F, Fig. 5) as well as relaxed/unwound dsDNA (lanes p2–p4 Panels D & E, Fig. 6) in pellet fraction as a function of RAD52. Even in other conditions, namely, AMP-PNP/ATPγS/AMP or in the absence of any nucleotide cofactor, a similar trend is observed, where RAD52 protein renders RAD51-dsDNA complexes, aggregation prone (Figs. 5 &6). Protein (lanes p2–p4, Panels G-J, Fig. 5) as well as DNA (lanes p2–p4, Panels F-H, Fig. 6) analyses reveal the trend that complexes comprising of RAD51/RAD52/dsDNA were "aggregated" as a function of RAD52 concentration. We speculate that these complexes in fact, may represent "co-aggregates" as both proteins are known to interact with each other in these reaction conditions [38]. Interestingly, DNA analyses reveal that "co-aggregates" contained not only substrate DNA i.e. relaxed dsDNA, but also products of unwinding. Quantitative analysis of proteins as well as DNA in the gel assays revealed that in all conditions of nucleotide cofactors tested, RAD51 protein and dsDNA co-sedimented as a function of RAD52 concentration. This is evidenced by an increase in recovery of RAD51 protein as well as dsDNA in pellet fractions with a concomitant depletion of the same in supernatant fractions [Fig. 5K: Protein analysis (data shown only for pellet fractions) & (Fig. 6I: DNA analysis (only pellet fraction data is shown)].

### Salt (KCl) induced changes in aggregation of RAD51-DNA complexes

**Figure 7:**
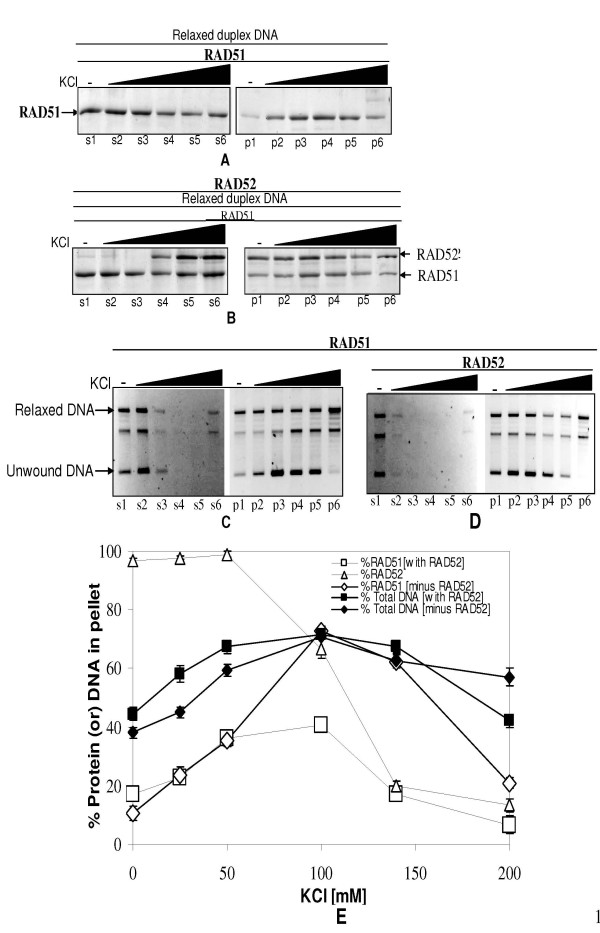
**KCl induced changes in the aggregation of RAD51-dsDNA complexes: protein and DNA analyses**. Relaxed φX174 DNA (30 μM) along with Topo I was incubated with RAD51 (3.0 μM) in presence of increasing concentration of KCl [0 mM, 25 mM, 50 mM, 100 mM, 140 mM, & 200 mM] with 1 mM ATP either in presence of RAD52 [2.0 μM] (Panel B: Protein analysis, Panel D:DNA analysis) or in its absence (Panel A: Protein analysis, Panel C:DNA analysis) in binding buffer R at 37°C for 12 minutes followed by centrifugation assay (Methods) and analyses of proteins by SDS-PAGE (Methods). Lanes s1–s6 and p1–p6 in all the Panels represent supernatant and pellet fractions for increasing concentrations of KCl respectively. RAD51 bands in pellet fraction is expressed as a fraction of the protein band intensities associated with the sum of supernatant and pellet fractions and plotted as a function of KCl concentration [(Open square: Panel E) in presence of RAD52] [(Open diamond: Panel E) in absence of RAD52)]. Similarly RAD52 protein is also represented in Panel E (open triangle). For DNA analysis, samples were deproteinized (Methods). Percentage of DNA (relaxed and unwound forms) in pellet fraction was expressed as a ratio of the total DNA (relaxed and unwound forms) in both pellet and supernatant fractions and plotted as a function of KCl concentration [(Filled square: Panel E) presence of RAD52 & (Filled diamond: Panel E) absence of RAD52].

In order to unravel any mechanistic link between RAD51-dsDNA aggregation and duplex unwinding, we probed the system by salt titration study. Moreover, we also tested whether salt induced aggregation of RAD51-dsDNA complexes would then obviate the need of RAD52 in the same. Centrifugation analysis of RAD51-DNA complexes as a function of varying KCl concentration in presence of ATP revealed interesting trends: RAD51-dsDNA aggregation increased in low salt (up to 100 mM KCl), followed by a drop at high salt regime (100–200 mM KCl) [(Fig. 7A: protein analysis & Fig. 7C: DNA analysis) (data shown only for pellet fractions)]. This trend was unaffected by the presence of RAD52 [(Fig. 7B: protein analysis) & (Fig. 7D: DNA analysis)]. Quantification of gel data revealed increase in RAD51 and dsDNA levels in pellet fractions as a function of salt concentration (up to 100 mM), followed by a decline of the same at higher salt concentration (100–200 mM) (Fig. 7E). Concomitantly, supernatant fractions revealed reciprocal trend. On the contrary, under same reaction conditions, RAD52 protein showed a different trend: RAD52 protein that was highly aggregated to begin with (without salt), disaggregated with the addition of salt. Salt induced disaggregation of RAD52 ensued from > 50 mM KCl onwards, and reached completion at about 140 mM KCl (Figs. 7B &7E).

### Salt (KCl) induced changes in dsDNA unwinding by RAD51

**Figure 8:**
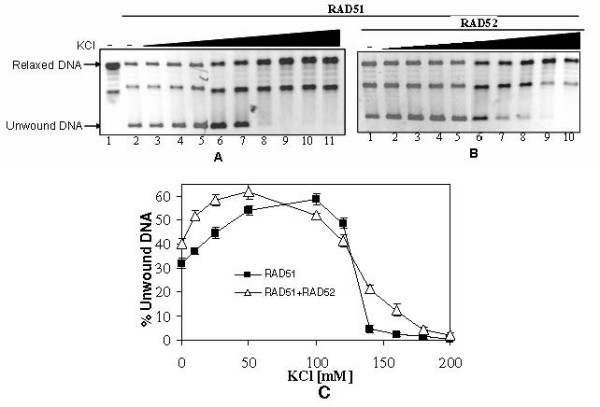
**KCl induced changes in dsDNA unwinding by RAD51**. Relaxed φX174 DNA (30 μM) along with Topo I was incubated with RAD51 [3.0 μM] in presence of increasing concentration of KCl [0 mM, 10 mM, 25 mM, 50 mM, 100 mM, 120 mM, 140 mM, 160 mM, 180 mM & 200 mM] with ATP either in presence of RAD52 [2.0 μM] (Panel B: lanes 1–10) or in its absence (Panel A: lanes 2–11). Lane 1 in Panel A represents no protein control. Unwound DNA band intensity was expressed as percentage of total DNA band intensities per lane and plotted as a function of KCl concentration (Panel C).

In an attempt to correlate salt (KCl) induced changes in aggregation of RAD51-dsDNA complexes with corresponding changes in DNA unwinding, we analyzed RAD51 mediated unwinding at varying concentrations of KCl. Quantitative analysis of gel assay revealed that unwinding was stimulated in low salt concentration (reaching up to 100–120 mM KCl), followed by a fall in the same at higher KCl concentration (> 100 mM KCl) (Fig. 8). In fact, by about 160 mM KCl, the unwinding level was barely detectable. By and large the effect of salt (KCl) on RAD51 mediated unwinding was very similar even in presence of RAD52. Consequently, the salt titration studies suggested that low concentration of salt (up to 100 mM) which is conducive for RAD51-dsDNA aggregation, in parallel, also resulted in an increase in dsDNA unwinding. Conversely KCl at high concentration (> 100 mM) led to decrease in aggregation as well as dsDNA unwinding. RAD52 protein in RAD52-RAD51-dsDNA complexes started disaggregating at concentrations of KCl greater than 50 mM (Figs. 7B &7E), thereby suggesting that changes in RAD51-dsDNA aggregation as well as dsDNA unwinding are relatively independent of RAD52 protein in the same regime of salt concentration. Accordingly, these experiments further suggest that process of dsDNA unwinding is mechanistically linked to the aggregation state of RAD51 in RAD51-dsDNA complexes.

**Figure 9:**
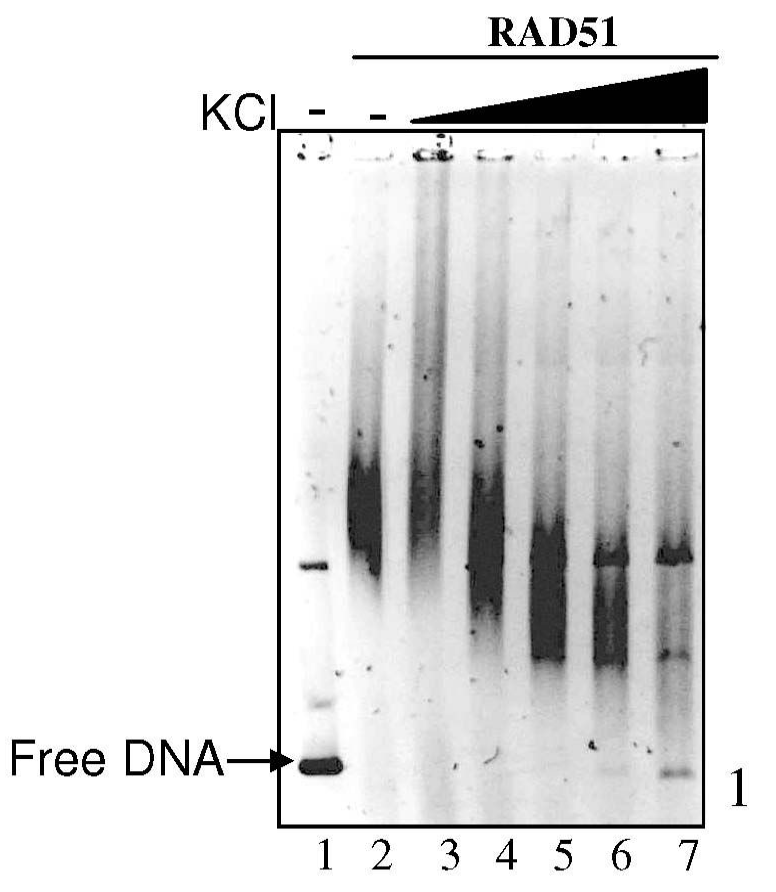
**Effect of KCl on RAD51 binding to dsDNA: gel shift assay**. Supercoiled φX174 DNA (30 μM) was incubated with RAD51 (3 μM) with ATP (1 mM) in binding buffer R at 37°C for 12 minutes in presence of increasing concentrations of KCl [0 mM, 25 mM, 50 mM, 100 mM, 140 mM & 200 mM] (lanes 2–7), followed by gel-shift analyses (Methods). Lane 1 represents no protein control.

In order to test whether enhanced aggregation as well as dsDNA unwinding observed in parallel at low salt (up to 100 mM KCl) was also accompanied by any changes in RAD51 binding to dsDNA, we analyzed the samples by gel-shift assays. Since the observed effects of salt were independent of RAD52 (as described above), we analyzed RAD51-dsDNA complexes as a function of KCl in the absence of RAD52. Gel-shift analyses revealed that mobility of protein-DNA complexes increased as a function of salt, thereby suggesting that RAD51 binding to dsDNA was reduced in the presence of salt. At high concentration of salt [200 mM] the protein-DNA complexes appeared to have dissociated, giving rise to the release of free dsDNA (lane 7, Fig. 9).

## Discussion

Our current study primarily shows that conditions which favour efficient aggregation of RAD51-dsDNA complexes such as presence of RAD52 or inclusion of salt in reaction leads to enhanced DNA unwinding in presence of suitable nucleotide cofactors. RAD52 by itself does not mediate DNA unwinding (Fig. 2G). Therefore, the only plausible mechanism by which RAD52 stimulates RAD51 mediated unwinding is by affecting RAD51-DNA interactions. The high aggregation prone nature of RAD52 protein is well established by Dynamic Light Scattering and Electron Microscopy studies [35,37-40] and also centrifugation assays performed in the present study (Panel B, Fig. 5). RAD51 on the other hand, is largely a soluble protein in its native state (Panel D, Fig. 5). We speculate that aggregated form of RAD52 through its large binding surface acts as a scaffold and sequesters RAD51-dsDNA complexes in smaller volume and thereby enhances RAD51 reactivity by macromolecular crowding effects. Indeed, centrifugation assays suggest greater recovery of RAD51, RAD52 and unwound DNA in pellet fractions as a function of increasing RAD52 concentration (Figs. 5 &6).

RAD51 binding to dsDNA has been shown to be selectively disrupted by salt in the context of homologous pairing and DNA strand exchange [41]. In particular, high salt [200 mM KCl] is reported to significantly lower RAD51 binding to dsDNA [42]. Consequently, we used KCl as a tool to perturb RAD51-dsDNA/RAD52-RAD51-dsDNA aggregation and monitor the corresponding changes in unwinding profile. KCl at low concentrations (up to 100 mM) mimics RAD52 effect in aggregating RAD51-dsDNA complexes (Fig. 7) while stimulating DNA unwinding in parallel (Fig. 8). On the other hand, KCl at high concentrations (> 100 mM) leads to disaggregation of complexes and lowering of dsDNA unwinding. On the contrary, RAD52 protein in RAD52-RAD51-dsDNA complexes started disaggregating at concentrations of KCl greater than 50 mM (Figs. 7B &7E), thereby suggesting that changes in RAD51-dsDNA aggregation as well as dsDNA unwinding are relatively independent of RAD52 protein in the same regime of salt concentration. Therefore one can infer that low salt mimics RAD52 effect in enhancing RAD51-dsDNA aggregation as well as dsDNA unwinding. It is relevant to note that in the regime of salt concentration that is stimulatory for aggregation of RAD51-dsDNA complexes and dsDNA unwinding (up to 100 mM KCl), RAD51 binding to dsDNA is reduced (Fig. 9). Such reduction in protein binding leads to partially coated RAD51-dsDNA complexes, which in turn facilitates enhanced intermolecular aggregation. This interpretation is fully consistent with the observed properties of RecA-DNA complexes where incompletely coated filaments have been shown to aggregate better [43].

## Conclusion

We believe that increased aggregation of RAD51-dsDNA complexes in low salt conditions, just as in the presence of RAD52, facilitates macromolecular crowding effects wherein several RAD51 and dsDNA molecules are brought proximal to each other within RAD51-dsDNA aggregates. Such a favourable scenario facilitates dynamic intermolecular transfer of RAD51 between DNA molecules within such aggregates. RAD51 is able to sample relatively more dsDNA molecules because of such transient binding to DNA in contrast to sampling lesser dsDNA molecules when RAD51 and dsDNA complexes are not aggregated. One can easily envisage kinetic advantage in such aggregated complexes via steps of rapid protein transfer, since RAD51 protein mobility on dsDNA has been shown to be highly dynamic [34]. Presence of Topo I in unwinding assay used in our study irreversibly captures the events of unwinding (resulting from protein binding) even after the dissociation of bound protein. Therefore Topo-I assay cumulatively "scores" the events of unwinding that manifest as an increase in steady-state level of DNA unwinding in conditions that favour protein-DNA aggregation. In the current study we have shown that RAD52, specific interactor of RAD51 protein, facilitates RAD51-DNA aggregation by acting as a molecular scaffold. However, we expect that any known reagent (spermidine, polyvinyl alcohol etc.) that non specifically brings about macromolecular crowding of protein-DNA complexes leading to aggregation will also elicit better unwinding of DNA because the process of aggregation appears to be more relevant than the mode (specificity) of aggregation. We suppose that the enhanced unwinding of dsDNA associated with aggregated RAD51-dsDNA complexes is somewhat analogous to that of aggregated forms of RecA-DNA networks participating more actively in homology search steps [43,44]. Our future studies are aimed at exploring the details of such dynamic intermediates in RAD51 catalyzed reactions.

## Methods

φX174 supercoiled DNA substrate was obtained from New England Biolabs. Potassium Chloride salt was purchased from Sisco Research Laboratories Ltd. Competent HMS174 strain of *E. coli *DE3 cells carrying RecA mutation was purchased from Novagen. All nucleotide cofactors used in this study were purchased from Roche diagnostics. The integrity of ADP is assessed by Thin Layer chromatography (TLC) analysis [36] and it is free of ATP contamination. Ni-NTA agarose beads were purchased from Qiagen.

### Purification of bacterial recombinant proteins

#### Purification of RAD51

The RAD51 gene cloned in pET15b vector to overexpress N-terminus hexa histidine tagged protein was obtained from Hitoshi Kurumizaka, Japan and the protein was purified as per the following protocol described elsewhere [45]. The plasmid (pET-HsRad51) is transformed in HMS174 strain (F-recA1 hsdR (rK12- mK12+) (DE3) (Rif R)) of *E. coli *cells. This strain carries the RecA mutation in a K-12 background. The transformed cells are grown in 4 liters of LB medium containing 100 μg/ml ampicillin at 30°C till A_600 _0.6 and induced with 1 mM IPTG. The cells are harvested 4 hours post induction. The cell pellet is resuspended in 60 ml of lysis buffer T (50 mM Tris-HCl [pH 8.0], 0.5 M NaCl, 5 mM β mercaptoethanol, 10 mM imidazole, 10% glycerol) containing 0.2 mM PMSF. About 100 mg of lysozyme is added to the cell suspension and incubated on ice for 30 minutes. The cell suspension is sonicated on a Branson sonifier (constant output, continuous duty) by carrying out 15 sec × 8–10 bursts with 1 minute cooling in between in order to maintain the cell suspension at low temperature. The cell lysate is centrifuged at 25,000 r. p. m. for 45 minutes at 4°C. A high speed supernatant is incubated with 2.5 ml bed volume of Ni-NTA agarose beads (washed and equilibrated in lysis buffer) for 1 hour at 4°C. The beads are then packed into an Econo-column (Bio-Rad). The resin is washed with 150 ml of buffer containing 20 mM Tris-HCl [pH 8.0], 0.5 M NaCl, 60 mM imidazole, 10% glycerol. The protein was eluted with a (60–400) mM imidazole gradient in 20 mM Tris-HCl, 0.5 M NaCl, 10% glycerol. Fractions collected as 1 ml/tube are analyzed on 12% SDS-PAGE and those containing pure protein are pooled and dialyzed against the buffer containing 20 mM Tris HCl (pH 8.0), 50 mM KCl, 0.5 mM EDTA, 2 mM β mercaptoethanol, 10% glycerol. The RAD51 concentration is estimated by bradford's method with BSA as standard. Typical yield is about 10 mg of protein from 4 liter culture and the final concentration is about 30 μM. The RAD51 protein is found to be electrophoretically pure containing a single band as analyzed by 12% SDS-PAGE and is free of any nuclease or ATP regenerating system contamination.

#### Purification of RAD52

The RAD52 over expressing clone (pFB581) was obtained from Steve West (Cancer Research, UK). Protein is purified as per the following protocol described elsewhere [46]. The recombinant RAD52 has six histidine residues at its N-terminus. The overexpressing clone is transformed into HMS174 strain (F-recA1 hsdR (rK12- mK12+) (DE3) (Rif R)) of *E. coli *cells. This strain carries the RecA mutation in a K-12 background. Transformed cells are grown in 4 litres of LB medium containing 100 μg/ml ampicillin at 37°C till A_600 _is 0.6. The cells are induced to overexpress RAD52 with 1 mM IPTG. The cells were harvested after 4 hours of induction. The cells are resuspended in 60 ml of lysis buffer T (20 mM Tris-HCl [pH 8.0], 0.5 M NaCl, 10% glycerol, 0.02% triton × 100) containing 0.2 mM PMSF, 5 mM imidazole and lysed by sonication as described previously. The cell lysate is centrifuged at 25,000 r. p. m. for 45 minutes at 4°C. The supernatant is mixed with 2.5 ml bed volume of Ni-NTA-agarose beads (washed and equilibrated in T buffer) for 1 hour at 4°C. The resin is washed sequentially with lysis buffer containing 25 mM and 50 mM imidazole respectively. The protein is eluted with a (50–500) mM imidazole gradient in T buffer. Fractions collected as 1 ml/tube are analyzed on 12% SDS-PAGE. Protein containing fractions were pooled and dialyzed against buffer B (20 mM Tris-HCl [pH 8.0], 1 mM EDTA, 0.5 mM DTT, 10% glycerol) containing 0.2 mM PMSF and 50 mM KCl. The dialyzed sample is loaded onto a 10 ml Q-sepharose column (equilibrated in buffer B). The column is washed with 100 ml of buffer B containing 50 mM KCl. RAD52 is eluted using a (50–500) mM KCl gradient in buffer B. Fractions containing pure RAD52 (as analyzed by 12% SDS-PAGE) are pooled, dialyzed against buffer containing 20 mM Tris HCl (pH 8.0), 50 mM KCl, 0.5 mM EDTA, 1 mM DTT, 10% glycerol, 0.05 mM PMSF. RAD52 concentration is estimated by bradford's method with BSA as standard. Typical yield is about 30 mg of protein from 4 liter culture and the final concentration is about 30 μM. The RAD52 protein is found to be electrophoretically pure containing a single band as analyzed by 12% SDS-PAGE and is free of any nuclease or ATP regenerating system contamination.

### Purification of Drosophila Topoisomerase I (Topo I)

The smallest active N-terminal truncated domain of Drosophila Topoisomerase I was cloned into pET-28a expression vector from a cDNA. The coding sequence was subcloned into Nco1/XhoI restriction sites in frame with the C-terminal hexa Histidine tag. The construct referred to as pET-NDH6 was provided by Dr. T. K. Kundu (JNCASR, Bangalore) and protein is purified as per the following protocol described [47]. pET-NDH6 is transformed into BL21 (DE3) cells and plated on LB plates conataining 50 μg/ml kanamycin and incubated overnight at 37°C. One average sized colony is innoculated in 0.5 litre LB medium conataining 50 μg/ml kanamycin at 37°C till A_600 _reached 0.5 and induced with 0.42 mM IPTG. The cells were shifted to 30°C and incubated for 5 hours post induction. The cells are then harvested by centrifugation at 7000 r. p. m. for 10 minutes at 4°C. The cell pellet is resuspended in 10 to 20 ml of lysis buffer T (50 mM Sodium phosphate [pH 7.0], 0.5 M NaCl, 15% (v/v) glycerol, 15 mM imidazole, 0.1% (v/v) NP-40) containing 0.2 mM PMSF and 10 mM Na_2_S_2_O_5 _and lysed by sonication as described previously. The cell lysate is centrifuged at 25,000 r. p. m for 45 minutes at 4°C. The high speed supernatant is added to 1 ml (bed volume) of Ni-NTA resin (washed and equilibrated with buffer T) and incubated for 3 hours in the cold room on a rocking platform. The resin is loaded onto a disposable 20-ml polypropylene column by gravity flow in a cold room. The column is washed 3 times with 10 ml of cold lysis buffer T. Protein is eluted in three fractions, each time with 1 ml of elution buffer (lysis buffer T containing 0.5 M imidazole) after discarding the void volume (the first 350 μl). Fractions are analyzed on 12% SDS-PAGE. Protein fractions are pooled and dialyzed against 2 litres of buffer (25 mM HEPES [pH 7.6], 0.1 mM EDTA, 10% (v/v) glycerol, 50 mM NaCl, 0.01% NP-40, 0.5 mM Dithiothreitol (DTT), 0.2 mM PMSF, 5 mM Na_2_S_2_O_5_) for 2 hours at 4°C. Dialysis is repeated against 1 liter storage buffer T (10 mM potassium HEPES, pH [7.6], 0.1 mM EDTA, 50 mM NaCl, 0.01% (v/v) NP-40, 50% (v/v) glycerol, 10 mM β mercaptoethanol) containing 0.2 mM PMSF and 1 μg/ml leupeptin. The concentration is estimated using bradford's reagent with BSA as standard. The typical yield is about 1.5 to 2.0 mg protein in 1.0 to 2.0 ml from 500 ml culture. The activity of the protein is assayed by relaxing 0.5 μg supercoiled plasmid DNA with different dilutions of protein.

### DNA Relaxation assay by Drosophila Topo I

φX174 supercoiled DNA (91 μM nucleotides) was incubated with Drosophila Topo I (130 nM) in buffer R containing 30 mM Tris-HCl [pH 7.6], 1 mM Dithiothreitol (DTT), 1 mM MgCl_2 _at 37°C for 1 hour. An aliquot of the relaxed DNA was checked on a 0.8% agarose gel. It is to be noted that all the purified recombinant proteins and buffers used in this study are free of contamination from ATP regenerating system as assessed by TLC analysis.

### Topological assay to measure unwinding of relaxed dsDNA by RAD51

Indicated amounts of protein (see legends) was incubated with topologically relaxed φX174 DNA (30 μM nucleotides) containing Topo I (43 nM) in a 20 μL reaction volume in buffer R containing 30 mM Tris-HCl [pH 7.6], 1 mM Dithiothreitol (DTT), 1 mM MgCl_2 _without or with any of 1 mM ATP/ATPγ S/ADP/AMP-PNP/AMP. Reaction mixtures were incubated for 12 minutes at 37°C and then deproteinised with SDS (1% final) and proteinase K (0.5 mg/ml) for 30 minutes at 37°C. Samples were resolved on 0.8% agarose gels run in TAE buffer at room temperature (RT) (~25°C) at 78 V for 6 hours. DNA species were stained with ethidium bromide (0.5 mg/ml) for 30 minutes. After destaining in water for 20 minutes, gels were analyzed in a gel documentation system (Bio-Rad) and quantified using Image J software. It is to be noted that the Topo I relaxation efficiency varied a little from experiment to experiment as revealed by varying residual supercoiled DNA present in relaxed DNA preparation. Whenever residual supercoiled DNA was present in relaxed DNA sample, that background signal (percentage of supercoiled DNA per total DNA intensity) was subtracted from the percentage unwound DNA recovered in each reaction lane. Therefore % Unwound DNA plotted on Y-axes in our graphs represent background-corrected values. A data point in each graph is an average across three independent experiments where the standard error bars depict the variation between experiments. Statistical significance was assessed according to a two-tailed t-test: P < 0.05.

### DNA binding by gel-shift assays

φX174 supercoiled DNA was incubated with various concentrations of RAD51 in binding buffer R at 37°C for 12 minutes in the presence or absence of RAD52, without or with any of 1 mM ATP/ATPγS/ADP/AMP-PNP/AMP. Samples were run on a 0.8% agarose gel in TAE buffer at 78V for 6 hrs at RT. DNA and DNA-protein complexes were visualized post staining with ethidium bromide (0.5 mg/ml) and destaining with water. For analyzing protein composition of gel-shifted complexes, the region of the gel (0.8% low melting agarose) containing gel-shifted complex was excised and electrophoresed on 12% SDS-PAGE followed by Coomassie staining.

### Centrifugation assay

Reaction mixtures containing relaxed φX174DNA and RAD51/RAD52 proteins (see legends for details) were incubated in binding buffer R at 37°C for 12 minutes. Samples were centrifuged with eppendorf centrifuge at 16, 100 g for 10 minutes at RT. The supernatant and pellet fractions were separated and heated in Laemmli buffer at 90°C for 10 minutes and analyzed by SDS/PAGE (12% acrylamide), followed by Coomassie staining. For DNA analysis, supernatant and pellet fractions were deproteinised by SDS/Proteinase K treatment, run on 0.8% agarose gel and analyzed following ethidium bromide staining. The thickness of agarose gel used for running supernatant fractions is two fold greater than that used for pellet fractions in order to accommodate greater sample volume. Therefore comparisons between supernatant and pellet sample profiles were achieved better by quantification and not visually. Intensity of DNA was quantified using Image J software. Each experiment is performed thrice and data points are statistically analyzed as explained earlier for topological assay. In both topological as well as centrifugation assays we do not find significant variation between experiments, thereby testifying that *in vitro *reaction conditions were reliably executed.

## Abbreviations

RAD51: human Rad51; RAD52: human Rad52; HR: Homologous Recombination; ssDNA: single stranded DNA; dsDNA: double stranded DNA; Topo I: Topoisomerase I; ATP: Adenosine triphosphate; ADP: Adenosine diphosphate; AMP: Adenosine monophosphate; ATPγS: Adenosine 5'-O-(3-thiotriphosphate); AMP-PNP: adenosine 5'-[β, γ-imido] triphosphate; KCl: Potassium Chloride; BSA: Bovine Serum Albumen; TLC: Thin Layer Chromatography; A_600_: Absorbance at 600 nm; P MSF: Phenyl Methyl Sulphonyl Fluoride.

## Authors' contributions

KB carried out all experimental work, acquired, analyzed and interpreted data. NK participated in drafting the manuscript and data interpretation. AK performed TLC analysis to check the integrity of ADP, RAD51 and RAD52. BJ supervised the overall progress of this project.

## References

[B1] West SC (2003). Molecular views of recombination proteins and their control. Nat Rev Mol Cell Biol.

[B2] Sung P, Krejci L, Van Komen S, Sehorn MG (2003). Rad51 recombinase and recombination mediators. J Biol Chem.

[B3] Zaitseva EM, Zaitsev EN, Kowalczykowski SC (1999). The DNA Binding Properties of *Saccharomyces cerevisiae *Rad51 Protein. J Biol Chem.

[B4] Benson FE, Stasiak A, West SC (1994). Purification and characterization of the human Rad51 protein, an analogue of E. coli RecA. EMBO J.

[B5] Prasad TK, Yeykal CC, Greene EC (2006). Visualizing the Assembly of Human Rad51 Filaments on Double-stranded DNA. J Mol Biol.

[B6] Shinohara A, Ogawa T (1995). Homologous recombination and the roles of double strand breaks. Trends Biochem Sci.

[B7] Baumann P, West SC (1998). Role of the human RAD51 protein in homologous recombination and double-stranded-break repair. Trends Biol Sci.

[B8] Sung P, Robberson DL (1995). DNA strand exchange mediated by a RAD51-ssDNA nucleoprotein filament with polarity opposite to that of RecA. Cell.

[B9] Sung P (1994). Catalysis of ATP-dependent homologous DNA pairing and strand exchange by yeast RAD51 protein. Science.

[B10] Baumann P, Benson FE, West SC (1996). Human Rad51 protein promotes ATP-dependent homologous pairing and strand transfer reactions in vitro. Cell.

[B11] Wu AM, Bianchi M, DasGupta C, Radding CM (1983). Unwinding associated with synapsis of DNA molecules by recA protein. Proc Natl Acad Sci USA.

[B12] Murayama Y, Kurukawa Y, Mayanagi K, Iwasak H (2008). Formation and Branch migration of Holliday junctions mediated by eukaryotic recombinases. Nature.

[B13] De Zutter JK, Knight KL (1999). RAD51 and RecA proteins show significant differences in cooperative binding to single-stranded DNA. J Mol Biol.

[B14] Tombline G, Fishel R (2002). Biochemical characterization of the human RAD51 protein. I. ATP hydrolysis. J Biol Chem.

[B15] Stark JM, Hu P, Pierce AJ, Moynahan ME, Ellis N, Jasin M (2002). ATP hydrolysis by mammalian RAD51 has a key role during homology-directed DNA repair. J Biol Chem.

[B16] Gupta RC, Bazemore LR, Golub EI, Radding CM (1997). Activities of human recombination protein. Proc Natl Acad Sci USA.

[B17] Chi P, Van Komen S, Sehorn MG, Sigurdson S, Sung P (2006). Roles of ATP binding and ATP hydrolysis in human Rad51 recombinase function. DNA Repair.

[B18] Morrison C, Shinohara A, Sonoda E, Yamaguchi-Iwai Y, Takata M, Weichselbaum RR, Takeda S (1999). The essential functions of human Rad51 are independent of ATP hydrolysis. Mol Cell Biol.

[B19] Krejci L, Damborsky J, Thomsen B, Duno M, Bendixen C (2001). Molecular dissection of interactions between Rad51 and members of the recombination-repair group. Mol Cell Biol.

[B20] Milne GT, Weaver DT (1993). Dominant negative alleles of RAD52 reveal a DNA repair/recombination complex including Rad51 and Rad52. Genes & Dev.

[B21] Shen Z, Cloud KG, Chen DJ, Park MS (1996). Specific interactions between the human Rad51 and human Rad52 proteins. J Biol Chem.

[B22] Krejci L, Song B, Bussen W, Rothstein R, Mortensen UH, Sung P (2002). Interaction with Rad51 is indispensable for recombination mediator function of Rad52. J Biol Chem.

[B23] Liu Y, Maizels N (2000). Co-ordinated response of mammalian Rad51 and Rad52 to DNA damage. EMBO Rep.

[B24] Gasior SL, Olivares H, Ear U, Hari DM, Weichselbaum R, Bishop DK (2001). Assembly of RecA-like recombinases: distinct roles for mediator proteins in mitosis and meiosis. Proc Natl Acad Sci USA.

[B25] Miyazaki T, Bressan DA, Shinohara M, Haber JE, Shinohara A (2004). *In vivo *assembly and disassembly of Rad51 and Rad52 complexes during double-strand break repair. EMBO J.

[B26] Sugawara N, Wang X, Haber JE (2003). In vivo roles of Rad52, Rad54, and Rad55 proteins in Rad51-mediated recombination. Mol Cell.

[B27] Wolner B, Van Komen S, Sung P, Peterson CL (2003). Recruitment of the recombinational repair machinery to a DNA double-strand break in yeast. Mol Cell.

[B28] Benson FE, Baumann P, West SC (1998). Synergistic actions of Rad51 andRad52 in recombination and DNA repair. Nature.

[B29] Shinohara A, Ogawa T (1998). Stimulation by Rad52 of yeast Rad51-mediated recombination. Nature.

[B30] New JH, Sugiyama T, Zaitseva E, Kowalczykowski SC (1998). Rad52 protein stimulates DNA strand exchange by Rad51 and replication protein A. Nature.

[B31] Sung P (1997). Function of yeast Rad52 protein as a mediator between replication protein A and the Rad51 recombinase. J Biol Chem.

[B32] Sugiyama T, Kowalczykowski SC (2002). Rad52 protein associates with replication protein A (RPA)-single stranded DNA to accelerate Rad51 mediated displacement of RPA and presynaptic complex formation. J Biol Chem.

[B33] New JH, Kowalczykowski SC (2002). Rad52 protein has a second stimulatory role in DNA strand exchange that complements Replication protein-A function. J Biol Chem.

[B34] Granéli A, Yeykal CC, Robertson RB, Greene EC (2006). Long-distance lateral diffusion of human Rad51 on double-stranded DNA. Proc Natl Acad Sci USA.

[B35] Kim HK, Morimatsu K, Norden B, Ardhammar M, Takahashi M (2002). ADP stabilizes the human Rad51-single stranded DNA complex and promotes its DNA annealing activity. Genes Cells.

[B36] Norman GA, Follett MJ, Hector DA (1974). Quantitative thin-layer chromatography of ATP and the products of its degradation in meat tissue. J chromatogr.

[B37] Van Dyck E, Hajibagheri NMA, Stasiak A, West SC (1998). Visualisation of Human Rad52 Protein and its complexes with RAD51 and DNA. J Mol Biol.

[B38] Navadgi VM, Shukla A, Vempati RK, Rao BJ (2006). DNA mediated disassembly of RAD51 and RAD52 proteins and recruitment of RAD51 to ssDNA by RAD52. FEBS J.

[B39] Stasiak AZ, Larquet E, Stasiak A, Muller S, Engel A, Van dyck E, West SC, Egelman EH (2000). The human Rad52 protein exists as a heptameric ring. Curr Biol.

[B40] Jackson D, Dhar K, Wahl JK, Wold MS, Borgstahl GE (2002). Analysis of the human replication protein A: Rad52 complex: evidence for crosstalk between RPA32, RPA70, Rad52 and DNA. J Mol Biol.

[B41] Sigurdsson S, Trujillo K, Song BW, Stratton S, Sung P (2002). Basis for Avid Homologous DNA Strand Exchange by Human Rad51 and RPA. J Biol Chem.

[B42] Liu Y, Stasiak AZ, Masson JY, Mcllwraith MJ, Stasial A, West SC (2004). Conformational changes modulate the activity of Human RAD51 Protein. J Mol Biol.

[B43] Chow SA, Radding CM (1985). Ionic inhibition of formation of RecA nucleoprotein networks blocks homologous pairing. Proc Natl Acad Sci USA.

[B44] Tsang SS, Chow SA, Radding CM (1985). Networks of DNA and RecA protein are intermediates in homologous pairing. Biochemistry.

[B45] Kurumizaka H, Aihara H, Kagawa W, Shibata T, Yokoyama S (1999). Human Rad51 amino acid residues required for Rad52 binding. J Mol Biol.

[B46] Navadgi VM, Dutta A, Rao BJ (2003). Human Rad52 facilitates three-stranded pairing that follows no strand exchange: A novel pairing function of the protein. Biochemistry.

[B47] Swaminathan V, Hari Kishore A, Febitha KK, Kundu TK (2005). Human Histone Chaperone Nucleophosmin Enhances Acetylation-Dependent Chromatin Transcription. Mol Cell Biol.

